# Supplementary material for: Impaired mitochondrial oxidative metabolism in skeletal progenitor cells leads to musculoskeletal disintegration
Source: Nat Commun. 2022 Nov 11;13:6869. doi: 10.1038/s41467-022-34694-8 (PMC9652319; doi:10.1038/s41467-022-34694-8)

Impaired mitochondrial oxidative metabolism in skeletal progenitor cells leads to musculoskeletal disintegration

Chujiao Lin, Qiyuan Yang, Dongsheng Guo, Jun Xie, Yeon-Suk Yang, Sachin Chaugule, Ngoc DeSouza, Won-Taek Oh, Rui Li, Zhihao Chen, Aijaz A John, Qiang Qiu, Lihua Julie Zhu, Matthew Greenblatt, Sankar Ghosh, Shaoguang Li, Guangping Gao, Cole Haynes, Charles P Emerson, Jae-Hyuck Shim

Supplementary information:

- Supplementary Figures 1-12
- Supplementary Tables 1-2
- uncropped scans of immunoblots

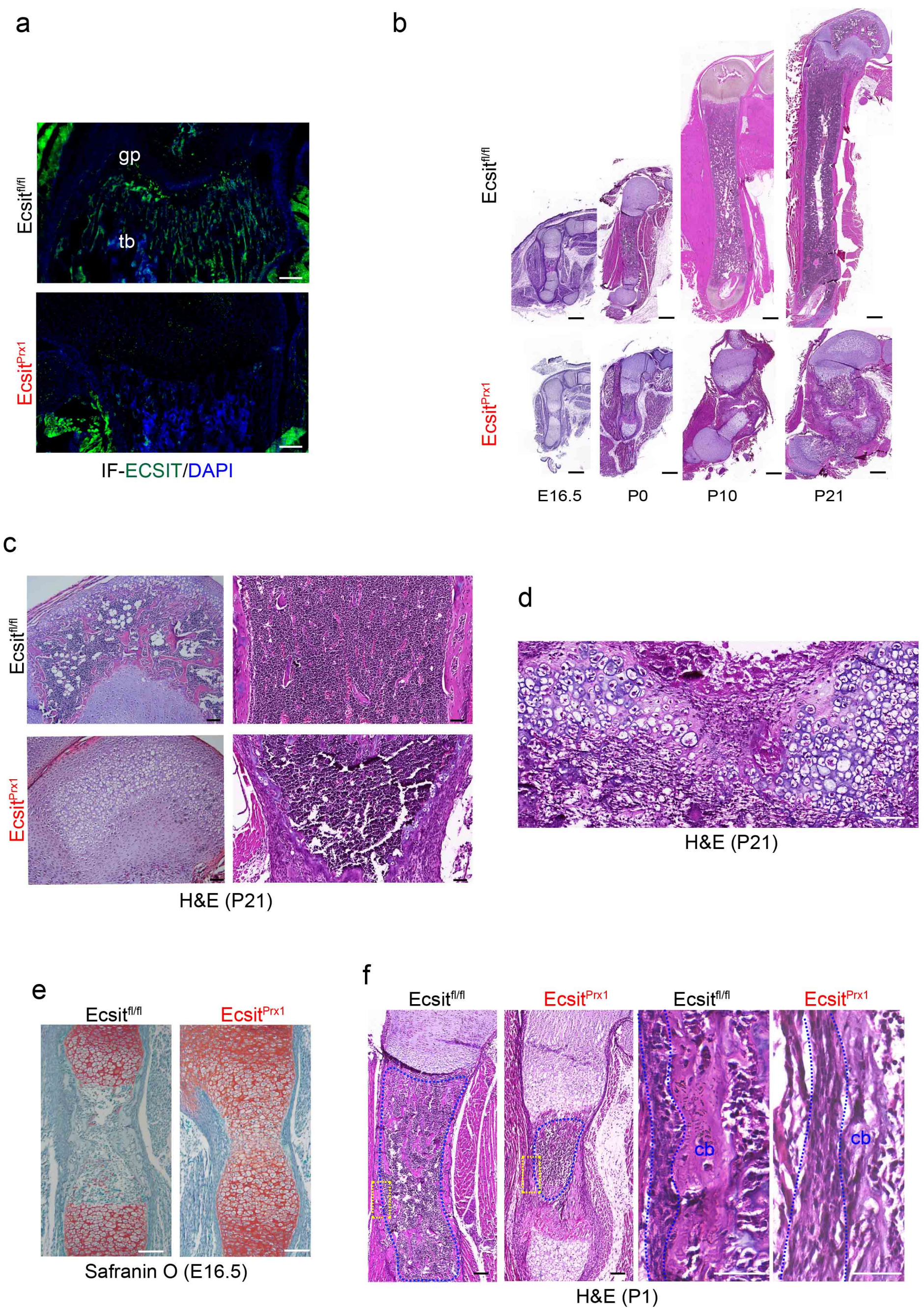

Supplementary Figure 1: Characterization of skeletal phenotypes in *Ecsit*^{Prx1} mice.

a. Immunofluorescence for ECSIT in the femurs of 2 month old *Ecsit*$^{fl/fl}$ and P21 *Ecsit*^{Prx1} mice, demonstrating deletion efficiency of *Ecsit* in Prx1-lineage cells. gp: growth plate, tb: trabecular bone. Scale bars = 100 μm. **b**. H&E staining of longitudinal sections of *Ecsit*$^{fl/fl}$ and *Ecsit*^{Prx1} femurs at different ages. Scale bars = 500 μm. **c, d**. H&E staining of longitudinal sections of P21 *Ecsit*$^{fl/fl}$ and *Ecsit*^{Prx1} femurs (**c**). High magnification of H&E staining shows cartilage elements in the fractured area of P21 *Ecsit*^{Prx1} femur (**d**). Scale bars = 100 μm. **e**. Safranin O staining of longitudinal sections of E16.5 *Ecsit*$^{fl/fl}$ and *Ecsit*^{Prx1} femurs. Scale bars = 100 μm. **f**. H&E staining of longitudinal sections of P1 *Ecsit*$^{fl/fl}$ and *Ecsit*^{Prx1} femurs. Blue lines and yellow boxes indicate bone marrow areas and high magnification images of diaphyseal periosteum and cortical bone. Scale bars = 100 μm. Data are representative of three independent experiments.

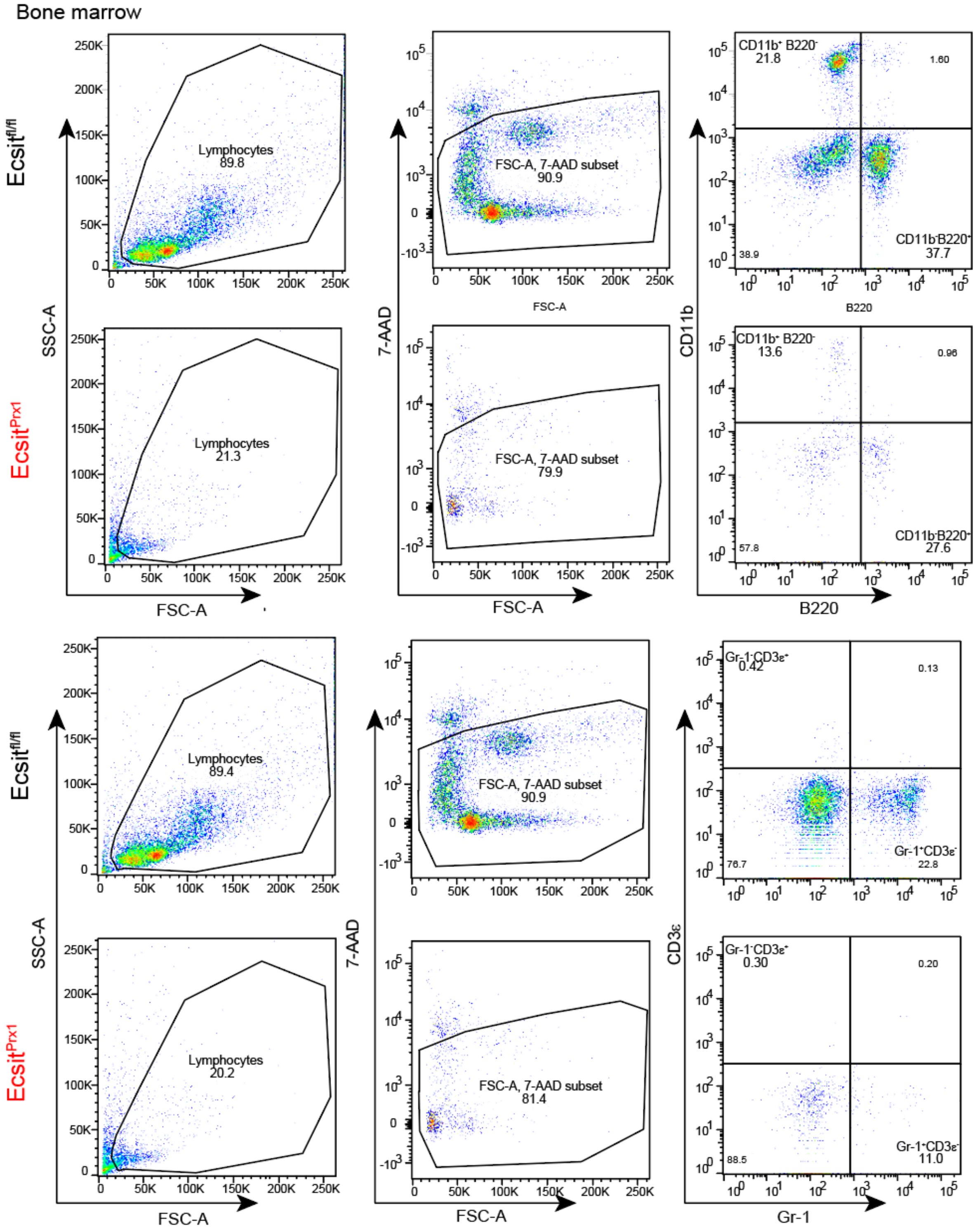

Supplementary Figure 2: Characterization of bone marrow cells in *Ecsit^{Prx1}* mice.
Flow cytometry analysis showing the frequency of immune cells (CD11b$^+$ and Gr-1$^+$ myeloid cells, CD3ε$^+$ T lymphocytes and B220$^+$ B lymphocytes) in the bone marrow of P21 *Ecsit$^{fl/fl}$* and *Ecsit^{Prx1}* mice (n = 4 – 7). Data are representative of three independent experiments.

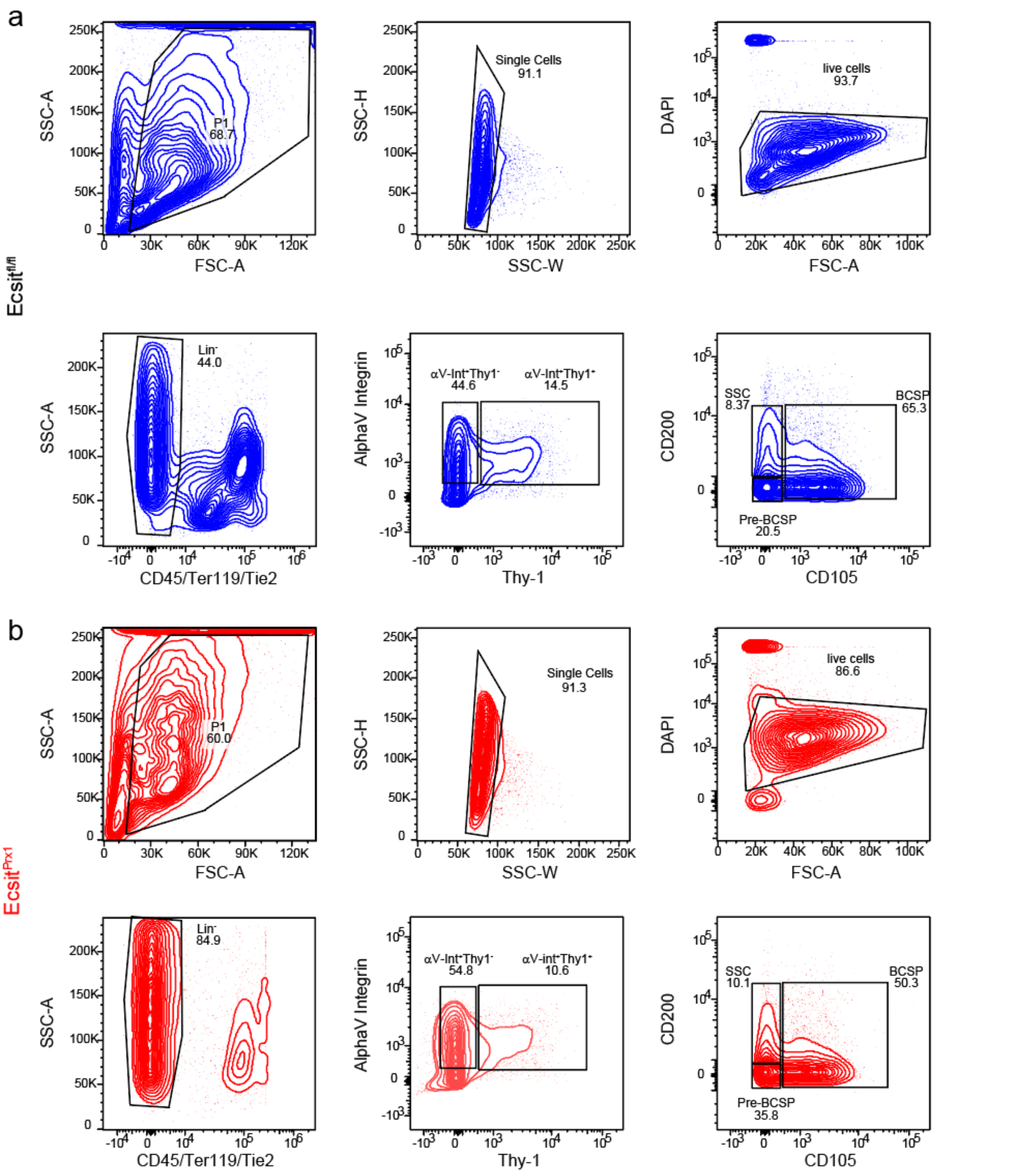

Supplementary Figure 3: Characterization of skeletal cell populations in *Ecsit*^{Prx1} mice.
P0 *Ecsit*$^{fl/fl}$ (blue) and *Ecsit*^{Prx1} (red) limbs were digested and subjected to flow cytometry analysis to demonstrate the gating strategy of SSCs (CD45$^-$Ter119$^-$Tie2$^-$αV-Int$^+$Thy1$^-$6C3$^-$CD105$^-$CD200$^+$), pre-BCSPs (CD45$^-$Tr119$^-$Tie2$^-$ αV-Int $^+$Thy1$^-$6C3$^-$CD105$^-$CD200$^-$), and BCSPs (CD45$^-$Ter119$^-$Tie2$^-$αV-Int$^+$Thy1$^-$ 6C3$^-$CD105$^+$) (n = 3). Data are representative of three independent experiments.

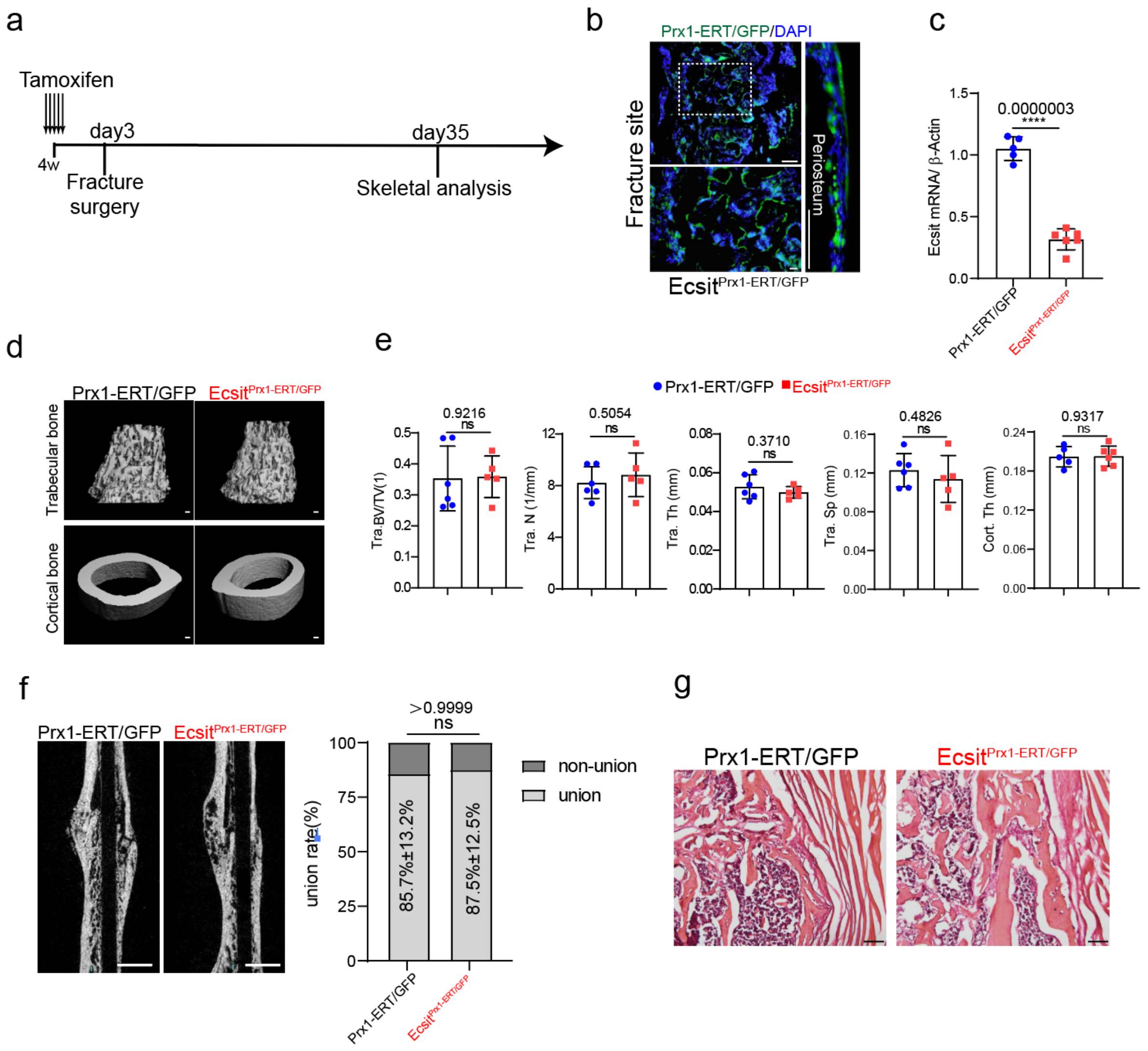

Supplementary Figure 4: Bone fracture healing and accrual occur normally in tamoxifen-treated *Ecsit*$^{Prx1\text{-}ERT/GFP}$ mice.

a. Diagram of the study and treatment methods. Fracture surgery was performed on the right femurs of 4 week old *Prx1-ERT/GFP* or *Ecsit*$^{Prx1\text{-}ERT/GFP}$ mice 3 days after tamoxifen treatment. One month later, skeletal analyses were performed. **b**. Fluorescence microscopy shows tamoxifen-induced expression of GFP in the fractured sites of *Ecsit*$^{Prx1\text{-}ERT/GFP}$ mice. Periosteal formation occurs normally in the fractured sites. Scale bars: 50 μm. **c**. mRNA levels of *Ecsit* in the tibia were assessed by RT-PCR (n = 5). **d, e**. MicroCT analysis of bone mass in non-fractured femurs of tamoxifen-treated *Prx1-ERT/GFP* or *Ecsit*$^{Prx1\text{-}ERT/GFP}$ mice. 3D-reconstruction images (**d**) and relative quantification of trabecular bone mass and cortical bone thickness (**e**, *Prx1-ERT/GFP*, n = 6; *Ecsit*$^{Prx1\text{-}ERT/GFP}$, n = 5) are displayed. **f**. MicroCT analysis shows fracture healing of tamoxifen-treated *Prx1-ERT/GFP* or *Ecsit*$^{Prx1\text{-}ERT/GFP}$ femurs. Representative sagittal views of fractured sites (**left**) and the relative quantification of fracture union rates (**right**, n = 5 - 6) are displayed. Scale bars: 100 μm. **g**. H&E staining of longitudinal sections of fractured sites of treated femurs, demonstrating normal fracture healing. Scale bars: 100 μm. A two-tailed unpaired Student's t-test for comparing two groups (**c, g**; error bars, SD of biological replicates). Data were presented as mean ± SD. Data are representative of three independent experiments.

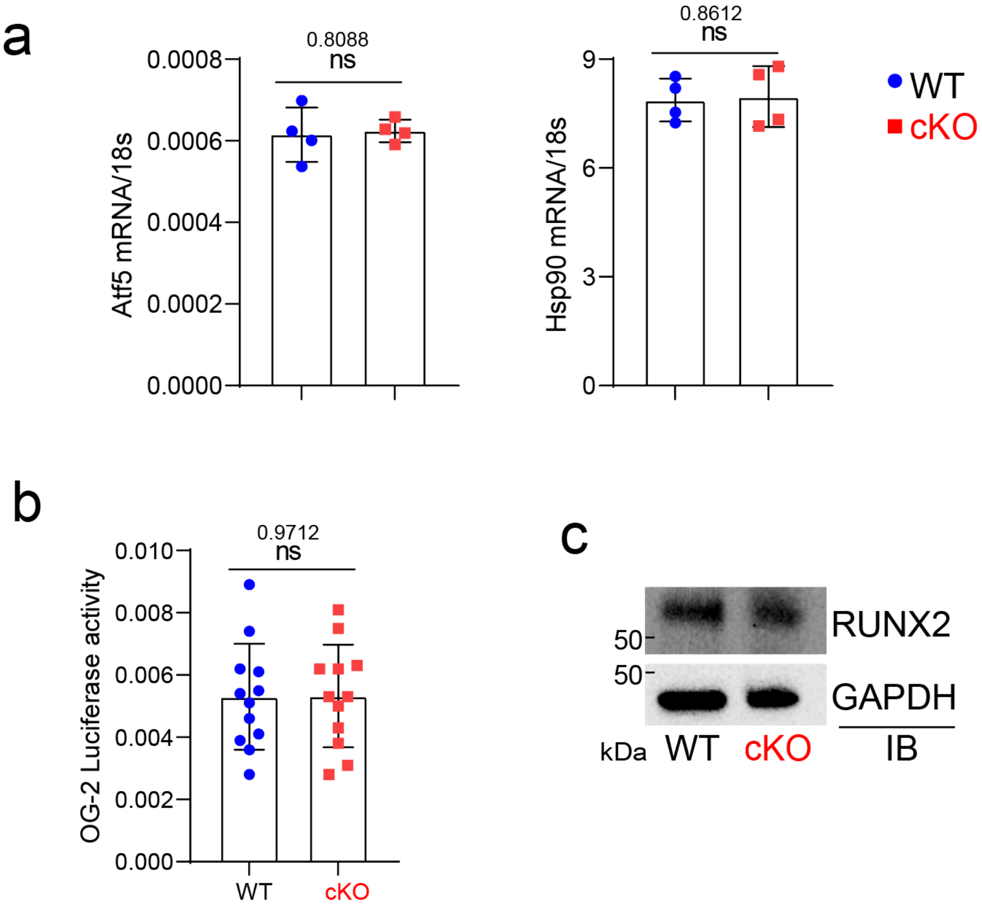

Supplementary Figure 5: RUNX2 functions normally in *Ecsit*^{Prx1} skeletal progenitors.
a. mRNA levels of mitochondrial biogenesis genes, Atf5 and Hsp90, in WT or cKO skeletal progenitors were assessed by RT-PCR (n = 4). Data were presented as mean ± SD. **b**. WT or cKO skeletal progenitors were transfected with the RUNX2-responsive reporter gene (OG2-Luc) and *Renilla*. 48 hours later, luciferase activity was measured using a luciferase assay and normalized *Renilla* (n = 12). Data were presented as mean ± SD. **c**. Immunoblot analysis shows protein levels of RUNX2 in WT or cKO skeletal progenitors. A two-tailed unpaired Student's t-test for comparing two groups (**a, b**; error bars, SD of biological replicates). Data are representative of three independent experiments.

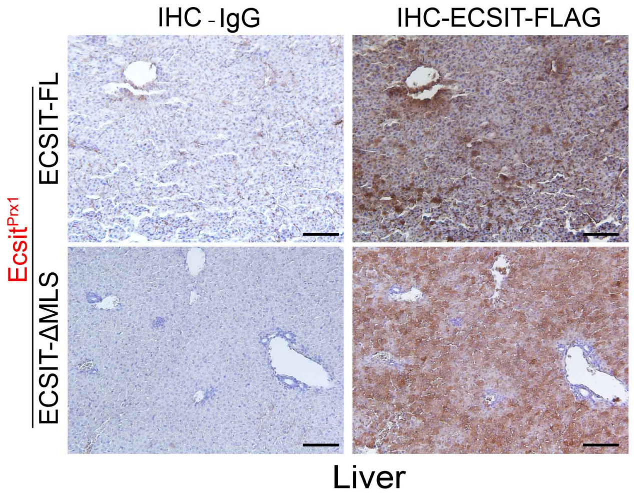

Supplementary Figure 6: Characterization of AAV9-treated *Ecsit*^{Prx1} mice.
A single dose of 2 x 10^{11} GCs of rAAV9 carrying FLAG-tagged ECSIT proteins (FL, ΔMLS) was injected into P1 *Ecsit*^{Prx1} neonates via facial vein and expression of FLAG-ECSIT proteins in P21 *Ecsit*^{Prx1} liver was determined by immunohistochemistry for FLAG. Scale bars = 75 μm. Data are representative of three independent experiments.

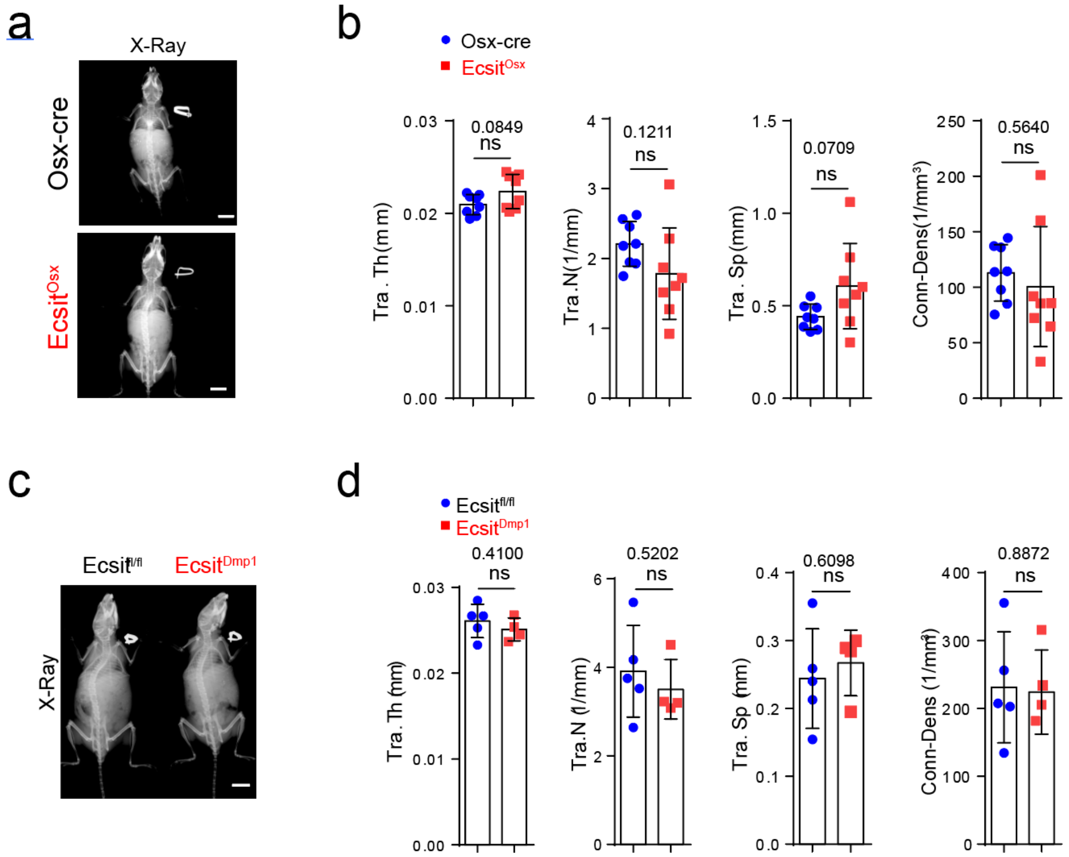

Supplementary Figure 7: Characterization of skeletal phenotypes in *Ecsit*^{Osx} and *Ecsit*^{Dmp1} mice.

Skeletal analyses of 2 month old *Ecsit*^{Osx} and *Ecsit*^{Dmp1} mice were performed using X-radiography of whole body (**a, c**) and microCT of the femurs showing the quantification of trabecular bone mass and cortical bone thickness (**b, d**). *Osx-cre* mice were used as a control group for *Ecsit*^{Osx} mice. Scale bars = 20 mm. A two-tailed unpaired Student's t-test for comparing two groups (**b, d**; error bars, data were presented as mean ± SD.). Data are representative of three independent experiments.

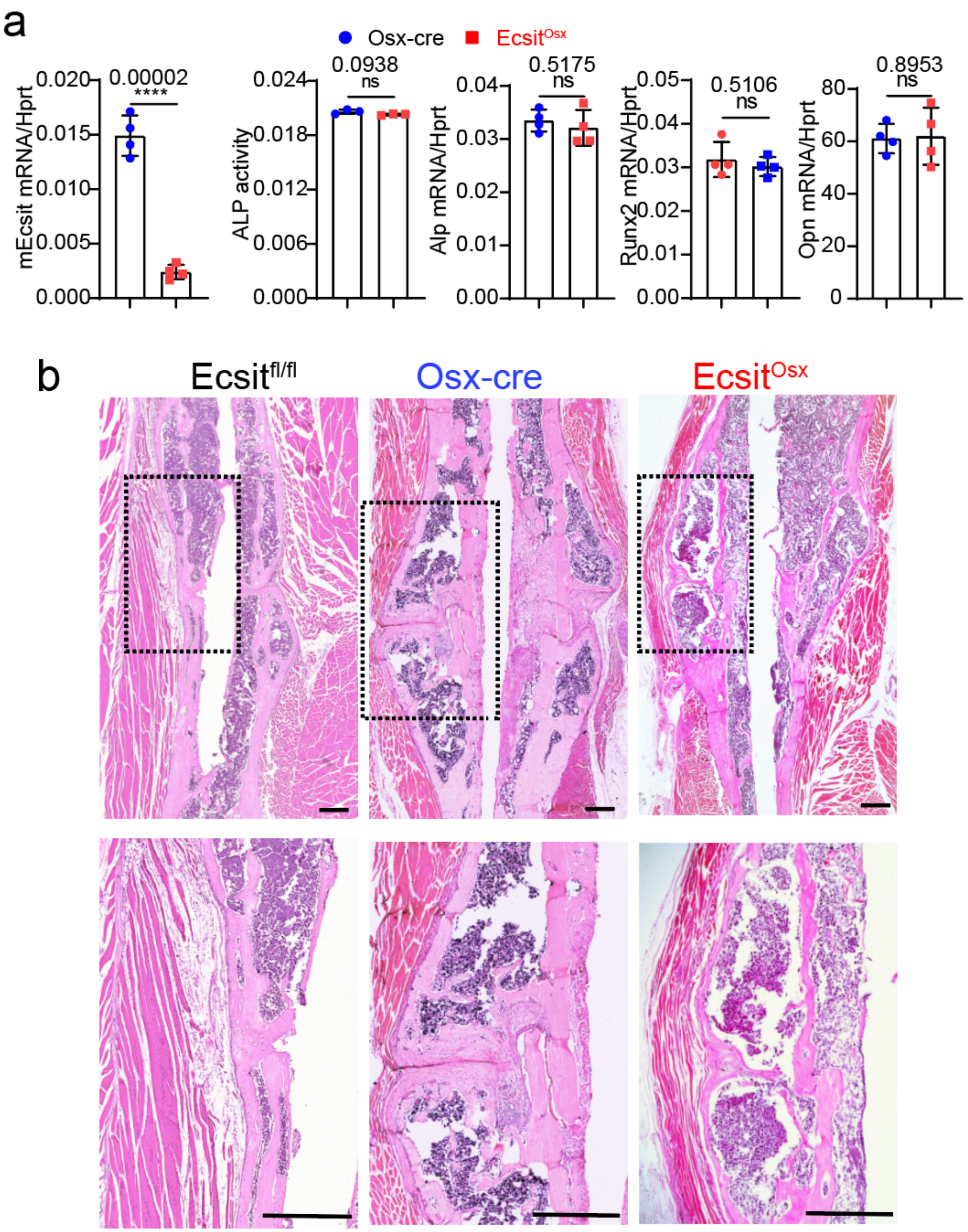

Supplementary Figure 8: Bone fracture healing occurs normally in *Ecsit*^{Osx} mice.

a. Osteoprogenitors were isolated from *Osx-cre* and *Ecsit*^{Osx} calvaria and cultured under osteogenic conditions. mRNA levels of *Ecsit* and osteogenic genes (n = 4 from four independent experiments) and alkaline phosphatase activity (ALP, n = 3) were assessed for osteogenic differentiation activity. **b.** H&E staining of longitudinal sections of fractured sites in 10 week old *Osx-cre*, *Ecsit*^{Osx}, and *Ecsit*$^{fl/fl}$ femurs (n = 5). Scale bars = 200 mm. Boxes indicate the fracture sites. A two-tailed unpaired Student's t-test for comparing two groups. Data were presented as mean ± SD.

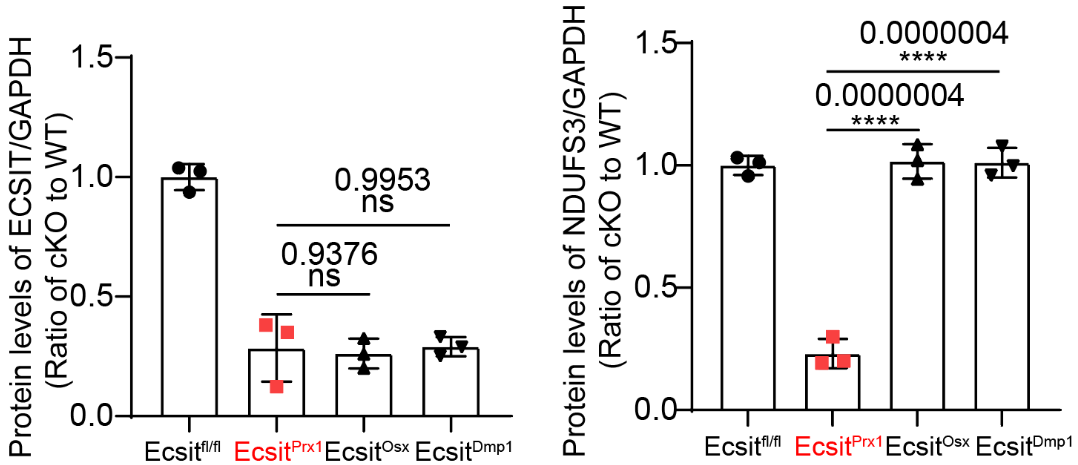

Supplementary Figure 9: Comparison of protein levels of ECSIT or NDUFS3 in *Ecsit$^{fl/fl}$*, *Ecsit^{Prx1}*, *Ecsit^{Osx}*, and *Ecsit^{Dmp1}* osteoblast-lineage cells. Protein levels of ECSIT and NDUFS3 in skeletal progenitors (*Ecsit^{Prx1}*), oseoprogenitors (*Ecsit^{Osx}*), and osteoblasts (*Ecsit^{Dmp1}*) were assessed by immunoblotting. Band intensities were measured by densitometric analysis using ImageJ software and normalized to GAPDH (n = 3 from three independent experiments). An ordinary one-way ANOVA with Dunnett's multiple comparisons test for comparing four groups. Data were presented as mean ± SD.

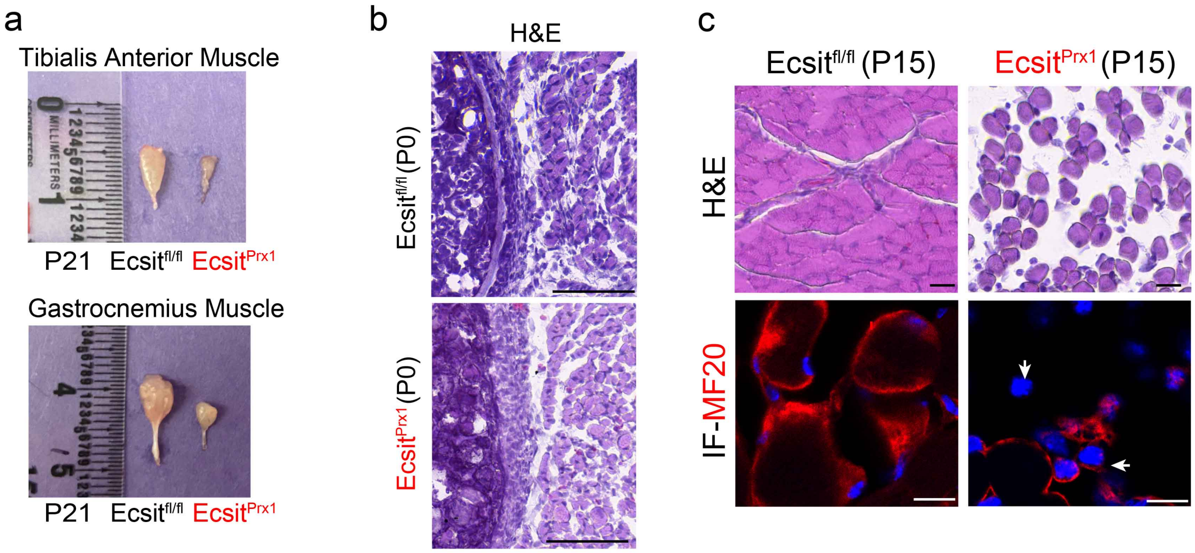

Supplementary Figure 10: *Ecsit^{Prx1}* mice display skeletal muscle atrophy.

a. Photographic images and of tibialis anterior (TA) and gastrocnemius (GA) muscles isolated from P21 *Ecsit^{Prx1}* and *Ecsit$^{fl/fl}$* mice. **b**. Transverse sections of muscles and bones isolated from P0 *Ecsit^{Prx1}* and *Ecsit$^{fl/fl}$* neonates were stained for H&E staining. Scale bars = 75 μm. **c**. High magnification of H&E staining (**top**) or immunostaining for MF20 (**bottom**) in P15 *Ecsit^{Prx1}* and *Ecsit$^{fl/fl}$* GA muscles. The same experiment was performed in **Figure 5d**. Scale bars = 25 μm. Data are representative of three independent experiments.

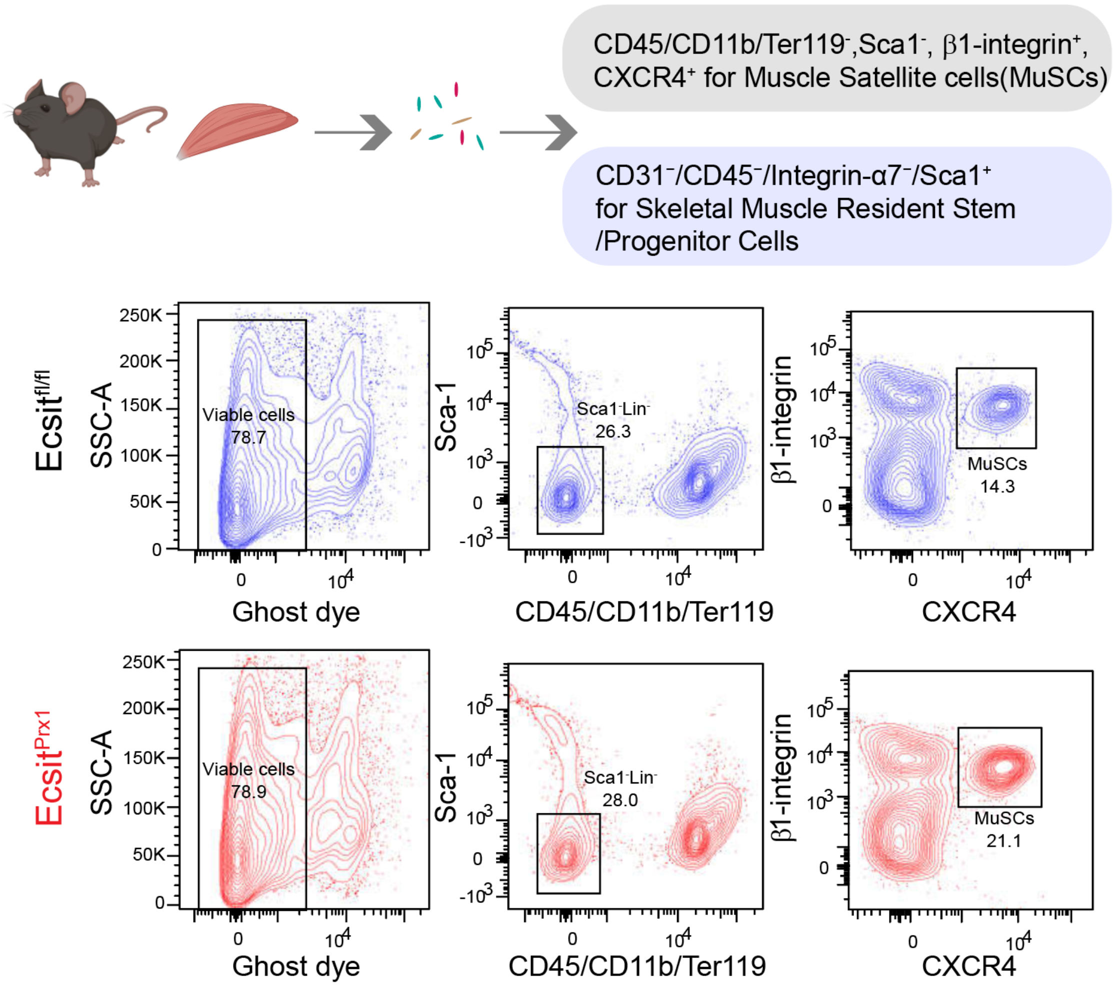

Supplementary Figure 11: Characterization of muscle cell populations in *Ecsit*^{Prx1} mice. Skeletal muscles from P10 *Ecsit*$^{fl/fl}$ (blue) and *Ecsit*^{Prx1} (red) mice were digested and subjected to flow cytometry analysis to demonstrate the gating strategy of MuSCs (Muscle Satellite Cells, Sca1$^-$β-integrin$^+$CXCR4$^+$CD45$^-$CD11b$^-$TER119$^-$) and skeletal muscle resident stem/progenitor cells (CD31$^-$CD45$^-$integrin-α7$^-$Sca1$^+$) (n = 5 – 9). Data are representative of three independent experiments. Mouse diagram was created with biorender.com.

a

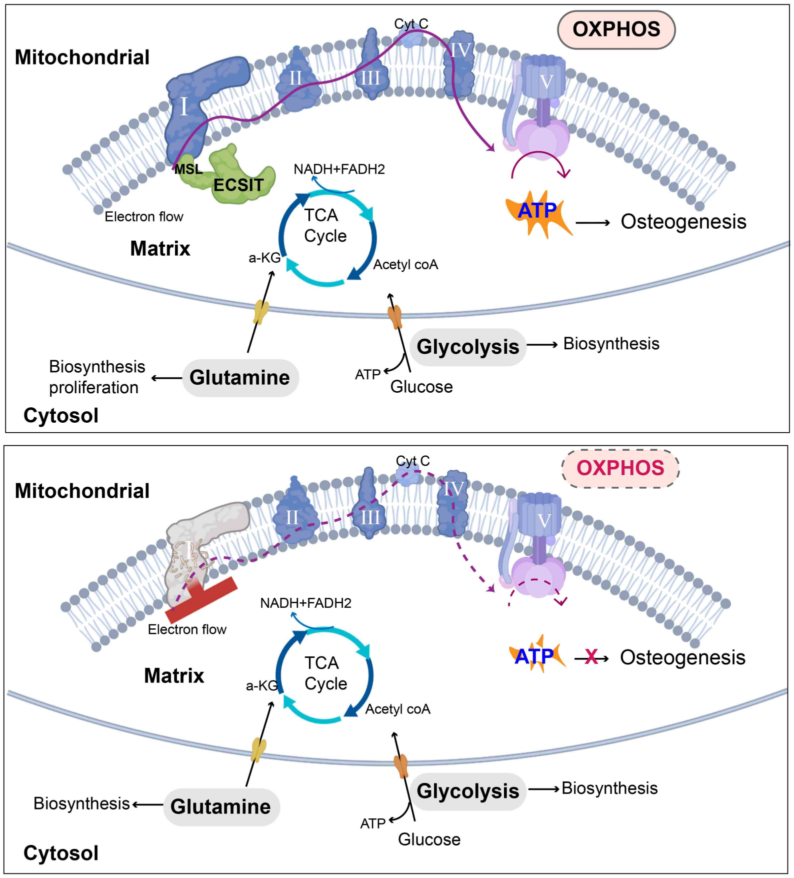

Mitochondrial
OXPHOS
Cyt C
I
II
III
IV
V
MSL
ECSIT
NADH+FADH2
Electron flow
TCA Cycle
ATP
Osteogenesis
Matrix
a-KG
Acetyl coA
Glycolysis
Biosynthesis
Biosynthesis proliferation
Glutamine
ATP
Glucose
Cytosol
Mitochondrial
OXPHOS
Cyt C
II
III
IV
V
NADH+FADH2
Electron flow
TCA Cycle
Matrix
a-KG
Acetyl coA
ATP
Osteogenesis
Glycolysis
Biosynthesis
Biosynthesis
Glutamine
ATP
Glucose
Cytosol

b

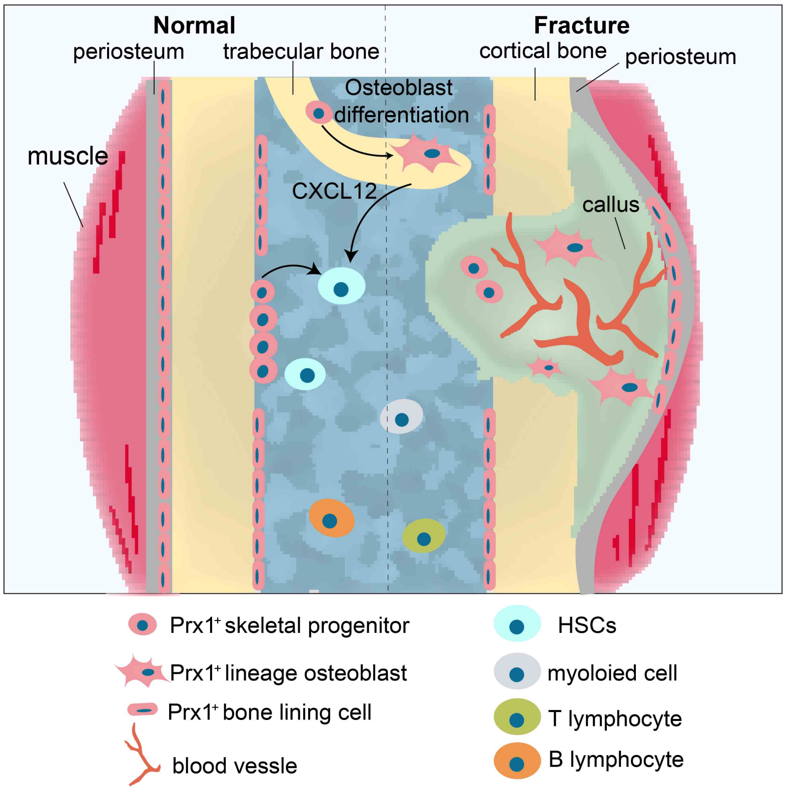

Normal
Fracture
periosteum
trabecular bone
cortical bone
periosteum
Osteoblast differentiation
muscle
CXCL12
callus
Prx1+ skeletal progenitor
Prx1+ lineage osteoblast
Prx1+ bone lining cell
blood vessle
HSCs
myoloied cell
T lymphocyte
B lymphocyte

Supplementary Figure 12: ECSIT controls mitochondrial OXPHOS and musculoskeletal integrity.

In the molecular levels, *Prx1*$^+$ skeletal progenitor-intrinsic function of ECSIT is required for mitochondrial OXPHOS by controlling CI assembly. ECSIT-deficient skeletal progenitors display defects in CI complex assembly, ATP production, and osteogenesis, whereas glucose and glutamine metabolism and tricarboxylic acid (TCA) cycle are largely intact in these cells. In the physiological levels, ECSIT-mediated osteogenesis is crucial for bone formation, regeneration, periosteal formation, and the bone marrow niche. ECSIT-deficiency in skeletal progenitors impairs osteogenic commitment and differentiation of skeletal progenitors, resulting in skeleton intrinsic effects (skeletal deformities and fracture healing failure) and skeleton extrinsic effects (impaired bone marrow niche maintenance and skeletal muscle atrophy). Diagram was created with biorender.com.

Supplementary Table 1: Phenotype summary of *Ecsit*^{Prx1} mice

Phenotype / Age	Cartilage expansion	Delay of primary ossification center	Delay of secondary ossification center	Skeletal deformity	Osteoporosis	Persistent nonunion	Spontaneous fracture	Bone marrow reduction
**E16.5**	+	+	NA	-	+	-	-	NA
**P0**	+	+	+	+	+	-	-	+
**P7**	+	+	+	+	+	+	+	+
**P14**	+	+	+	+	+	+	+	+
**P21**	+	+	+	+	+	+	+	+

NA: not-applicable

Supplementary Table 2: Sequences of primers, probes and gblocks

Gene	Forward	Reverse
Mouse *Ecsit*	AAGCTGTGGTTCACCCGATTC	GGGCAAAGACATCTGGTAGACAGT
Mouse *Alp*	CACAATATCAAGGATATCGACGTG[illegible]	ACATCAGTTCTGTTCTTCGGGTACA
Mouse *Runx2*	TACAAACCATACCCAGTCCCTGTT[illegible]	AGTGCTCTAACCACAGTCCATGCA
Mouse *Bsp*	CAGGGAGGCAGTGACTCTTC	AGTGTGGAAAGTGTGGCGTT
Mouse *Osx*	ATGGCGTCCTCTCTGCTTGA	GAAGGGTGGGTAGTCATTTG
Mouse *Ocn*	GCAGCACAGGTCCTAAATAG	GGGCAATAAGGTAGTGAACAG
Mouse *Hprt*	CTGGTGAAAAGGACCTCTCGAAG	CCAGTTTCACTAATGACACAAACG
Mouse *18s*	GTAACCCGTTGAACCCCATT	CCATCCAATCGGTAGTAGCG
Mouse *Myogenin*	GAGATCCTGCGCAGCGCCAT	CCCCGCCTCTGTAGCGGAGA
Mouse *Myf5*	AAACTCCGGGAGCTCCGCCT	GGCAGCCGTCCGTCATGTCC
Mouse *MyoD*	TCTGGAGCCCTCCTGGCACC	CGGGAAGGGGGAGAGTGGGG
Mouse *MRF4*	GTGGACCCCTACAGCTACAAACC	TGGAAGAAAGGCGCTGAAGAC
Mouse *Ndufv1*	CTTCCCCACTGGCCTCAAG	CCAAAACCCAGTGATCCAGC
Mouse *Tgf-β1*	TGATACGCCTGAGTGGCTGTCT	CACAAGAGCAGTGAGCGCTGAA
Human RPL13A	AACCTCCTCCTTTTCCAAGC	GCAGTACCTGTTTAGCCACGA
Human CKMα	ATGCCATTCGGTAACACCCAC	GCTTCTTGTAGAGTTCAAGGGTC
Human MYH8C	AATGCAAGTGCTATTCCAGAGG	ACAGACAGCTTGTGTTCTTGTT
Human MYOD	GCGGAACTGCTACGAAGG	AGGGCTCTCGGTGGAGAT
EGFP	AGCAAAGACCCCAACGAGAA	GGCGGCGGTCACGAA
Ecsit (FL)-Flag	ATGAGCTGGGTGCAGGTCAACTTGCTGGTGCGAAGCCTCTCTAGGGGCTG[illegible]<br>GAGGCCTCTGCAGGCCTGCCCTTTCAGGAACTCCCTTTGCTCAGGTGTCCC[illegible]<br>CAGGCTCTACGAGGCCTTCACTGTAGTGCAGCTACACACAAGGATGAGCCC[illegible]<br>GGTTGGTTCCTCGACCCCCGGAGCCCCAGAGGAAGCCCATCAAGGTTCCA[illegible]<br>AATGCATGAAGACTCGTTCAAGCCATCAGGAAACAGGGAGCGGGACAAGG[illegible]<br>AGCTTCCTGAACGCAGTGCGCAGCTTTGGGGCGCACAATGTGCGCAAGCG[illegible]<br>GCCACGTGGACTTCATCTACCTAGCACTGCGCAAGATGCCAGAGTTTGGTG[illegible]<br>GAGCGGGACTTGTCAGTATACAACCTGCTGCTGGATGTTTTCCCCAAGGAG[illegible]<br>CTTCCGGCCCCGCAACGTTATCCAGCGCATCTTCGTCCACTACCCACGGCA[illegible]<br>AGGAATGTGGGGTCGCAGTCCTGGAGCAGATGGAACGACACGGGGTCATG[illegible]<br>CAGCGCAGAGACAGAGTTCCTACTGATTCAGATATTCGGGCGCAAAAGTTA[illegible]<br>CCATGCTCAAGTTCCTGCGGATGAAGCTGTGGTTCACCCGATTCAAGAATA[illegible]<br>AACCCCTACCCAGTGCCCCGAGATCTTCCCCAGGACCCTTTGGACCTGGC[illegible]<br>GCTAGGCCTGCGACACATGGAGCCTGACCTCAGTGCTAAGGTCACTGTCT[illegible]<br>AGATGTCTTTGCCCAGTGACTCGACAGGCATGGAAGATCCCACACAGCCT[illegible]<br>ATTGTAGGAATCCAGAGTCCAGATCAGCAAGCTGCCCTGGCCCGCCACAA[illegible]<br>CATCCAGGCCTGTTTTTGTTGAGGGCCCCTTCCCTCTCTGGCTTCGTAATA[illegible]	

	GTGTCTACTATCACATCCTAAGAGCTGACCTGCCACCTCCTGAGGAAGAGA[illegible]<br>GTAGAGGAGATCCCAGAAGAATGGGAGCTGTACTACCCACAGAAGCTGGA[illegible]<br>TGGAATATTCAAGGAGTGGTTGGGACGACTATGAGTTTGACGTGGATGAAG[illegible]<br>ACAGAAGGCCCTGTCTTCGCCATGTGCATGGCTGGTGCCCATGATCAGGC[illegible]<br>CATTGATCAAGTGGATCCAAGGCTTGCAGGAGACCAACCCAACCCTGGCA[illegible]<br>ATCCCAGTGGTATTCCGCCTGGCCAGGTCCACGGGGGAGCTCCTGACAAC[illegible]<br>CAAGGCTGGAGGGACAGTCCCCTCCCCACAGTCCTCCCAAGGGGCCTGAG[illegible]<br>AGATGATGAGACCATTCAGGCAGAGCAGCAGCAGGGGCAAAGTGATTACA[illegible]<br>GATGACGACGATAAGTGA
Ecsit (∆MLS)-Flag	ATGAAGGATGAGCCGTGGTTGGTTCCTCGACCCCCGGAGCCCCAGAGGA<br>AGCCCATCAAGGTTCCAGCAATGCATGAAGACTCGTTCAAGCCATCAGGA<br>AACAGGGAGCGGGACAAGGCAAGCTTCCTGAACGCAGTGCGCAGCTTTG<br>GGGCGCACAATGTGCGCAAGCGCGGCCACGTGGACTTCATCTACCTAGC<br>ACTGCGCAAGATGCCAGAGTTTGGTGTGGAGCGGGACTTGTCAGTATAC<br>AACCTGCTGCTGGATGTTTTCCCCAAGGAGGTCTTCCGGCCCCGCAACG<br>TTATCCAGCGCATCTTCGTCCACTACCCACGGCAGCAGGAATGTGGGGT<br>CGCAGTCCTGGAGCAGATGGAACGACACGGGGTCATGCCCAGCGCAGA<br>GACAGAGTTCCTACTGATTCAGATATTCGGGCGCAAAAGTTACCCCATGC<br>TCAAGTTCCTGCGGATGAAGCTGTGGTTCACCCGATTCAAGAATATCAAC<br>CCCTACCCAGTGCCCCGAGATCTTCCCCAGGACCCTTTGGACCTGGCCA<br>AGCTAGGCCTGCGACACATGGAGCCTGACCTCAGTGCTAAGGTCACTGT<br>CTACCAGATGTCTTTGCCCAGTGACTCGACAGGCATGGAAGATCCCACAC<br>AGCCTCACATTGTAGGAATCCAGAGTCCAGATCAGCAAGCTGCCCTGGC<br>CCGCCACAACCCATCCAGGCCTGTTTTTGTTGAGGGCCCCTTCCCTCTCT<br>GGCTTCGTAATAAGTGTGTCTACTATCACATCCTAAGAGCTGACCTGCCA<br>CCTCCTGAGGAAGAGAAAGTAGAGGAGATCCCAGAAGAATGGGAGCTGT<br>ACTACCCACAGAAGCTGGACCTGGAATATTCAAGGAGTGGTTGGGACGA<br>CTATGAGTTTGACGTGGATGAAGTGACAGAAGGCCCTGTCTTCGCCATGT<br>GCATGGCTGGTGCCCATGATCAGGCAACATTGATCAAGTGGATCCAAGG<br>CTTGCAGGAGACCAACCCAACCCTGGCACAGATCCCAGTGGTATTCCGC<br>CTGGCCAGGTCCACGGGGGAGCTCCTGACAACTTCAAGGCTGGAGGGA<br>CAGTCCCCTCCCCACAGTCCTCCCAAGGGGCCTGAGGAAGATGATGAGA<br>CCATTCAGGCAGAGCAGCAGCAGGGGCAAAGTGATTACAAGGATGACGA<br>CGATAAGTGA

Uncropped scans of immunoblots (Supplementary Figure 5c)

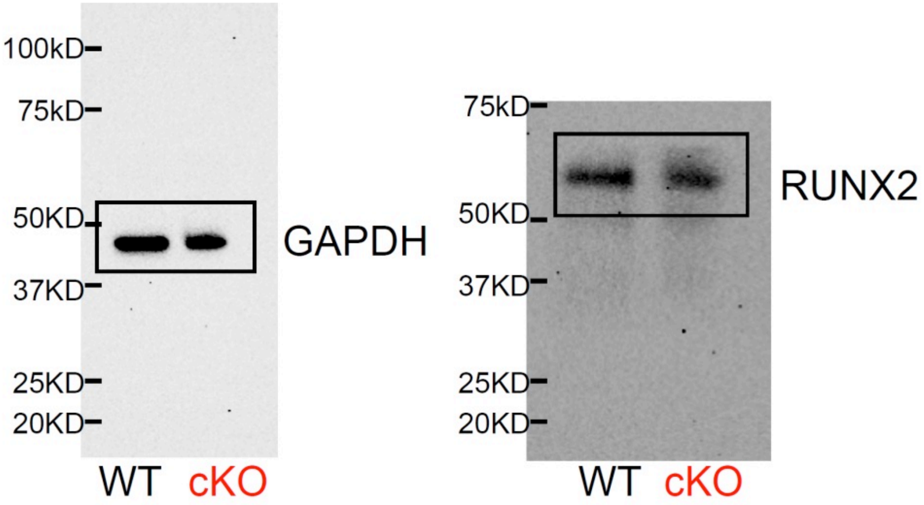

Supplement: Supplementary file 1 — Supplementary information [file 41467_2022_34694_MOESM1_ESM.pdf]
